# DNA-based culture-independent analysis detects the presence of group a streptococcus in throat samples from healthy adults in Japan

**DOI:** 10.1186/s12866-016-0858-5

**Published:** 2016-10-11

**Authors:** Tejaswini Kulkarni, Chihiro Aikawa, Takashi Nozawa, Kazunori Murase, Fumito Maruyama, Ichiro Nakagawa

**Affiliations:** 1 Department of Molecular Craniofacial Embryology, Graduate School of Medical and Dental Sciences, Tokyo Medical and Dental University, Tokyo, 113-8510 Japan; 2 Department of Microbiology, Graduate School of Medicine, Kyoto University, Kyoto, 606-8501 Japan

**Keywords:** Asymptomatic carriage, Culture-independent detection, *Emm*-type, Group A *Streptococcus*, Species-specific primers

## Abstract

**Background:**

Group A *Streptococcus* (GAS; *Streptococcus pyogenes*) causes a range of mild to severe infections in humans. It can also colonize healthy persons asymptomatically. Therefore, it is important to study GAS carriage in healthy populations, as carriage of it might lead to subsequent disease manifestation, clonal spread in the community, and/or diversification of the organism. Throat swab culture is the gold standard method for GAS detection. Advanced culture-independent methods provide rapid and efficient detection of microorganisms directly from clinical samples. We investigated the presence of GAS in throat swab samples from healthy adults in Japan using culture-dependent and culture-independent methods.

**Results:**

Two throat swab samples were collected from 148 healthy volunteers. One was cultured on selective medium, while total DNA extracted from the other was polymerase chain reaction (PCR) amplified with two GAS-specific primer pairs: one was a newly designed 16S rRNA-specific primer pair, the other a previously described V-Na^+^-ATPase primer pair. Although only 5 (3.4 %) of the 148 samples were GAS-positive by the culture-dependent method, 146 (98.6 %) were positive for the presence of GAS DNA by the culture-independent method. To obtain serotype information by *emm* typing, we performed nested PCR using newly designed *emm* primers. We detected the four different *emm* types in 25 (16.9 %) samples, and these differed from the common *emm* types associated with GAS associated diseases in Japan. The different *emm* types detected in the healthy volunteers indicate that the presence of unique *emm* types might be associated with GAS carriage.

**Conclusions:**

Our results suggest that culture-independent methods should be considered for profiling GAS in the healthy hosts, with a view to obtaining better understanding of these organisms. The GAS-specific primers (16S rRNA and V-Na^+^-ATPase) used in this study can be used to estimate the maximum potential GAS carriage in people.

**Electronic supplementary material:**

The online version of this article (doi:10.1186/s12866-016-0858-5) contains supplementary material, which is available to authorized users.

## Background

Group A *Streptococcus* (GAS; *Streptococcus pyogenes*), an extensively studied pathogen, causes mild and severe human diseases [1]. As an exclusively human pathogen, GAS is spread in the community through direct human-to-human transmission. GAS can also colonize a healthy host without any apparent clinical signs or symptoms [2, 3]. It has been reported that the rate of asymptomatic GAS carriage is high in children (2.5–32 %) and low in adults (1.5–4.9 %) [4–7]. Asymptomatic pathogen presence in people might be associated with reduced pathogen virulence and/or effective host immune responses against the pathogen [8]. The carriage state of the pathogen might lead to subsequent disease manifestation [9] and/or its metabolic diversification to cope with unfavorable conditions in the host such as harmful pH or nutrient depletion [7]. Therefore, investigating the GAS carriage state is a prerequisite for enhanced understanding of the population biology of GAS in terms of disease spread and pathogen survival in the community.

In clinical microbiology, standard cultivation methods are considered the ‘gold standard’ for microorganism detection because the phenotypic characterization of clinical isolates is important for determining the treatment directions, but such methods are laborious and time-consuming [10, 11]. Additionally, cultivation methods often fail to detect pathogens present in different physiological states such as viable but nonculturable (VBNC) in host niches [11, 12]. In contrast, by employing nucleic acid extracted directly from clinical samples, culture-independent methods provide rapid results for bacteria detection with high sensitivity and specificity [10, 11, 13, 14]. In particular, polymerase chain reaction (PCR)-based methods using species-specific primers targeting the 16S rRNA [15–21] or other conserved genes [11, 22–26] are used widely for detecting microorganisms in the fields of clinical and environmental microbiology.

The aim of the present study was to investigate the presence of GAS in throat swab samples from healthy adults in Japan using culture-dependent and culture-independent methods. We designed new GAS-specific 16S rRNA and *emm* primers and investigated the GAS carriage rate in swab samples from the throats of healthy adults. The specificities of these primers were tested against other *Streptococcus* species and their sensitivities were evaluated by serial dilution of the DNA extracts obtained from GAS cultures. The 16S rRNA primer pair was used for specific PCR detection of GAS from human throat swab samples in combination with the V-Na^+^-ATPase primer pair reported previously [27], while the newly designed *emm* primers were used to obtain *emm* sequence information to check the GAS serotypes.

## Methods

### Study design

The ethical committee of Tokyo Medical and Dental University approved this study. Throat swab samples were collected from October 2013 to June 2014 from students and staff from various departments in the Dental Faculty of the Tokyo Medical and Dental University, Tokyo, Japan (age range 20–60 years). All participants were volunteers who provided informed consent. Participants were considered to be healthy if they lacked any apparent clinical signs and symptoms such as sore throat or cold for at least 2 weeks before sample collection. Out of a total 165 participants, 17 with sore throat/throat pain or any kind of infection for unknown reasons were excluded from the final analysis (Additional file 1: Table S1).

### Sample collection

Two throat swabs were collected from the upper pharyngeal region of each volunteer (*n* = 148). One swab was dipped into 500 μl of sterile Trypticase soy broth (TSB; BBL Microbiology Systems, Cockeysville, Md.) for the culture-dependent study. The other swab was dipped into 600 μl of the sterile bead solution from the PowerSoil DNA isolation kit (MO BIO Laboratories, Carlsbad, CA), mixed for 20–30 s, and the resultant solution was stored at −80 °C prior to further extraction steps.

### GAS-specific primers

To design the GAS-specific primers, the 16S rRNA sequences from *Streptococcus* spp. were obtained from the Ribosomal Database Project (RDP) (http://rdp.cme.msu.edu/), and the potential PCR primer target regions were manually determined using a Probe Match algorithm. Among nine variable regions (V1–9) in the 16S rRNA sequences, the hypervariable 2 (V2) region is known to be one of the best target sequences for distinguishing *Streptococcus* spp. [15]. Therefore, we selected the V2 region and designed GAS-specific primers based on the alignment profiles made by a multiple sequence alignment program (MAFFT) with default parameters [28]. Primers for the putative sodium-V-ATPase subunit B (V-Na^+^-ATPase) region, as described by Hung et al. [27], was also used for GAS detection in the throat swab samples. To obtain the *emm*-type information from the total DNA extracted from the throat swabs, we adapted the nested PCR strategy for the *Vir* regulon (virulence regulon, recently referred to as *Mga*), which consists of a tandemly linked family of M proteins genes such as *fcrA*, *emm*, *enn*, *scpA* [29, 30]. *vir* primers, as described by Gardiner et al. [29], were used for the primary nested PCR. New *emm* primers were designed based on the alignment profiles of the untrimmed *emm* sequences in the Centers for Disease Control and Prevention (CDC) *emm* database (ftp://ftp.cdc.gov/pub/infectious_diseases/biotech/emmsequ/) by MAFFT with the default parameter. The nucleotide sequences of all the primer pairs are shown in Table 1.

**Table 1:** Primers used in this study

Primer	Nucleotide Sequence (5’-3’)	Amplicon size (bp)	Reference
16S rRNA	Fwd-AAGAGAGACTAACGCATGTTAGTAAT Rev-ATTTTCCACTCCCACCATCA	300	This study
V-Na^+^-ATPase	Fwd-GTCGATTTTGCCACGTACCG Rev -TGCATGGTCAACTCAATCATTTGC	121	Hung et al. 2012 [27]
Vir	SBR-AGACATGAGC**G**CAATGGCAAGTTTATCAAATGGTAATTTTTG VUF-AAACCGTATCTTTGACGCACTCGAGGACAATTTGCGAGATTAG	Variable	Gardiner et al. 1995 [29]
*emm*	Primer1 –TATT(C/G)GCTTAGAAAATTAA Primer 2– GCAAGTTCTTCAGCTTGTTT	Variable	CDC ^a^
*emm* conserved	Fwd-AATARACASTATTCGCTTAGAAAATTA Rev-GCTTAGTTTTCTTCTTTGCG	Variable	This study

*Abbreviations*: *Fwd* forward, *Rev* reverse, ^a^ *CDC* Centers for Disease Control and Prevention

For the SBR primer, G (shown in bold) replaces T in the original reference

### Primer specificity and sensitivity

To evaluate the specificities of the selected primers, 11 GAS strains (isolated from pharyngitis infections in infants with different GAS *emm* types) and 11 other non-GAS *Streptococcus* strains (listed in Additional file 1: Table S2) were incubated overnight in TSB at 37 °C. Genomic DNA extraction was performed using a DNeasy Blood and Tissue kit (Qiagen, Hilden, Germany) according to the manufacturer’s instructions, and the concentration of the extracted DNA was quantified using a Quant-iT PicoGreen dsDNA assay kit (Invitrogen, Carlsbad, USA). For primer specificity testing, PCR was performed in a 20 μl volume containing 2 ng of template DNA from the selected strains. *S. pyogenes* JRS4 DNA and sterile deionized water were used as the positive and negative controls, respectively. To check the primer sensitivities, PCRs were performed using serially diluted *S. pyogenes* JRS4 (M6) DNA with a concentration range from 0.625 fg to 6.25 ng, which corresponds to 0.3 to 3 × 10^6^ genome copies of GAS. All PCRs were performed using MightyAmp DNA polymerase (Takara, Tokyo, Japan).

### GAS detection methods

**Fig. 1:**
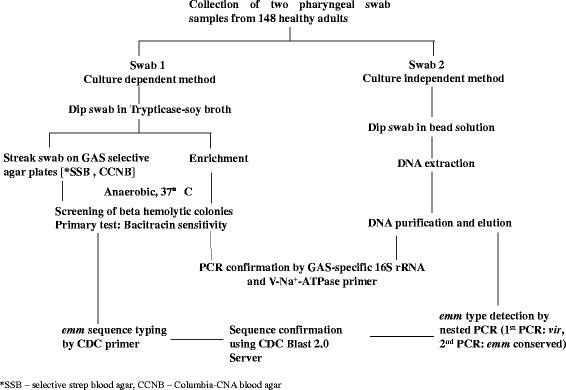
Schematic representation of the strategy used for GAS detection from the throat swabs of healthy people

Two methods, culture dependent and independent, were used to detect GAS in throat swabs from healthy adults (Fig. 1).

### Culture-dependent study

For the culture-dependent method, swabs were streaked onto the following two types of agar medium, supplemented with 5 % defibrinated sheep blood (Japan Biotest Laboratories, Tokyo, Japan): Selective Strep Agar, Modified#2 (SSB) (Acumedia Manufacturers Inc., Baltimore, MD) and Columbia-CNA agar (BD, Sparks, MD, USA). In addition to streaking, each throat swab dipped TSB broth was also added to 1.2 ml of fresh TSB for enrichment. Enriched cultures were spread onto SSB agar plates and incubated for a further 24 h. All cultivations were carried at 37 °C under anaerobic conditions using the AnaeroPack system (Mitsubishi Gas Chemical Co., Tokyo, Japan).

After 24 h incubation, colonies showing beta hemolysis were selected randomly and checked for bacitracin sensitivity by the disk diffusion method [2] using sterile paper discs impregnated with 0.04 U Bacitracin (Nacalai Tesque, Kyoto, Japan). Next, single colony lysates, prepared using Lyse-N-Go PCR reagent (Thermo Scientific, Rockford, IL, USA), were examined using GAS-specific 16S rRNA and V-Na^+−^ATPase primers, and GAS serotyping was performed using the CDC *emm* primers. The 25 μl PCR mixtures each contained 2.5 μl of 10 X Ex Taq buffer, 2 μl of dNTPs, 2 μl of a 10 μM primer mix, 0.125 μl of Ex Taq HS polymerase (Takara) and 1 μl of lysate. The PCR conditions used are listed in Additional file 1: Table S3.

### Culture-independent study

Total DNA was extracted from each throat swab-dipped bead solution using a PowerSoil DNA isolation kit (MO BIO) with some modifications. Briefly, 500 mg of RNA PowerSoil beads was added to the throat swab solution and mixed thoroughly. In addition to 60 μl of solution C1 from the kit, 450 μl of phenol:chloroform:isoamyl alcohol (25:24:1) (Nacalai Tesque) was added to each sample, and the rest of the procedure was performed according to the manufacturer’s instructions. A 100 μl aliquot of the extracted DNA solution was purified using Ethachinmate (Wako, Tokyo, Japan), and the pellet was then dissolved in 40 μl of Tris-EDTA buffer. The resultant samples were stored at −80 °C. The 50 μl PCR mixture contained 25 μl of 2 X MightyAmp buffer Ver.2 (Takara), 4 μl of a 10 μM primer mix, 1 μl of MightyAmp DNA polymerase (Takara) and 5 μl of template DNA. With the exception of the *emm* conserved PCR (2^nd^ nested PCR), 0.5 μl of bovine serum albumin (New England BioLabs, Beverly, MA, USA) and 5 μl of 5 M Betaine (Sigma-Aldrich, St Louis, MO, USA) were added to all other PCR mixtures. *S. pyogenes* JRS4 DNA and sterile deionized water were used as the positive and negative controls, respectively. The PCR conditions used for each primer pair are listed in Additional file 1: Table S3. PCR products were analyzed by electrophoresis on 2 % agarose gels with ethidium bromide staining.

For *emm* sequencing, 5 μl of each nested PCR product was checked using agarose gel electrophoresis. After confirmation of the desired amplicon, the remaining PCR product was run on a 2 % agarose gel and stained with SYBR safe DNA gel stain (Invitrogen). Amplicons ranging from 800 to 2000 bp were extracted from gels and purified using a NucleoSpin® Gel PCR Clean-Up kit (Macherey-Nagel, Düren, Germany), and then cloned into a pGEM-T vector (Promega, Medison, WI). The cloned PCR products were sequenced using M13 primers and the 3130 Genetic Analyzer (Applied Biosystems). The sequences obtained were referred to the CDC database to check the *emm* types (http://www2a.cdc.gov/ncidod/biotech/strepblast.asp).

## Results

### Primer sensitivity and specificity

**Fig. 2:**
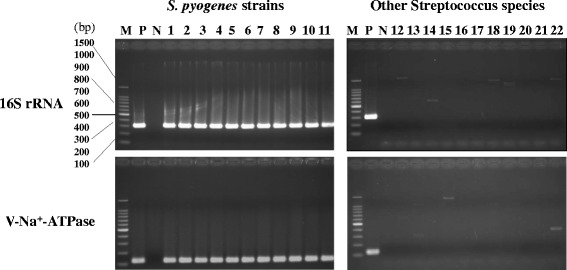
Primer specificity. Representative results of PCRs using GAS-specific 16S rRNA and V-Na^+^-ATPase primers. DNA templates (2 ng) from 11 different GAS strains and 11 other *Streptococcus* species were used for primer specificity testing. *S. pyogenes* JRS4 DNA and sterile deionized water were used for the positive and negative controls, respectively. Lane M, DNA marker; P, positive control; N, negative control; 1, *emm*1; 2, *emm*3; 3, *emm*4; 4, *emm*6; 5, *emm*11; 6, *emm*12; 7, *emm*28; 8, *emm*58; 9, *emm*75; 10, *emm*87; 11, *emm*89; 12, *S. salivarius*; 13, *S. sanguinis*; 14, *S. sobrinus*; 15, *S. suis*; 16, *S. mutans*; 17, *S. pneumoniae*; 18, *S. mitis*; 19, *S. dysgalactiae*; 20, *S. constellatus*; 21, *S. intermedius*; 22, *S. anginosus*

**Fig. 3:**
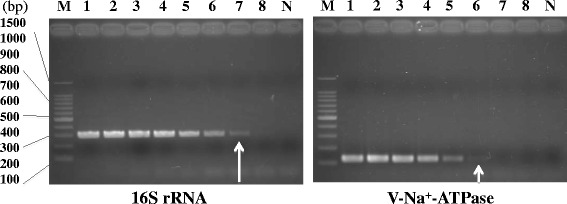
Primer sensitivity. Representative data for the detection limit of GAS-specific primers using MightyAmp DNA polymerase. *S. pyogenes* JRS4 DNA was serially diluted (6.25 ng to 0.625 fg DNA corresponds to 3 × 10^6^ to 0.3 genomic copies of GAS). Sterile deionized water was used for the negative control. Lane M, DNA marker; 1, 6.25 ng; 2, 625 pg; 3, 62.5 pg; 4, 6.25 pg; 5, 625 fg; 6, 62.5 fg; 7, 6.25 fg; 8, 0.625 fg; N, negative control. Arrows indicate the detection limits of the template DNA concentration

We used species-specific primers for detection of GAS from throat swab samples from healthy adults. The specificities of the 16S rRNA and V-Na^+^-ATPase primers were evaluated initially using bacterial DNA extracted from 11 different *emm*-type GAS strains and 11 other streptococcal species. Both primer pairs showed positive results for the GAS strains and did not cross-react with the other non-target streptococcal species (Fig. 2). The *emm* gene, which is a well-defined virulence GAS gene, encodes an antiphagocytic surface determinant, and *emm* typing is used widely in epidemiological surveillance of GAS [31]. To acquire *emm*-type information directly from the throat swabs, we designed new *emm* primers for nested PCR to enhance the efficiency of the subsequent analysis. However, nested *emm* PCR showed limited specificity in the samples we tested (data not shown). This result might be caused by cross reactivity with other streptococcal species and/or human DNA in the throat swab samples. The detection limit of all the primers was examined using serially diluted *S. pyogenes* JRS4 DNA (Fig. 3). Except for the V-Na^+^-ATPase primers, 16S rRNA, *vir* and the new *emm* primers detected 6.25 fg of GAS DNA. This result indicates that all the primers have sufficiently high sensitivities to detect even low abundance GAS populations in samples.

### GAS isolation by culture-dependent methodology

**Fig. 4:**
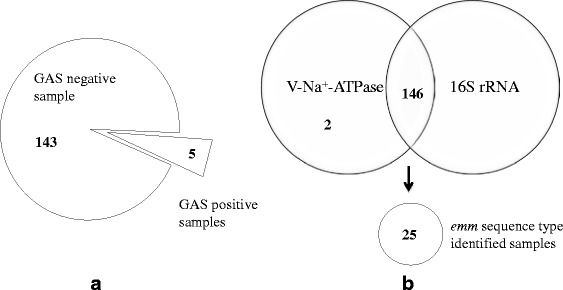
GAS detection from throat swab samples of healthy adults. **a** By the culture-dependent method, GAS was isolated from 5 of 148 swab samples. **b** By the culture-independent method, the presence of GAS DNA was PCR-confirmed for each sample by PCR with 16S rRNA and V-Na^+^-ATPase primers. *emm* sequence information was acquired from 25 samples. Additional *emm* type (detected in this study) information is shown in Additional file 1: Table S4

Selective culture media were used for better isolation of GAS from the swab samples. Additionally, we used strict anaerobic conditions during culture for effective recovery of GAS and for reducing aerobic commensal bacteria loads [32–34]. Among the 148 samples, GAS was isolated from five of them (3.4 %) (Fig. 4a). This rate is similar to those of earlier GAS carriage reports in healthy adults [6, 7]. All isolates were bacitracin sensitive and PCR positive using GAS-specific 16S rRNA and V-Na^+^-ATPase primers. The *emm* types were analyzed by *emm* sequence typing and by comparison with the sequences in the CDC *emm* database: tmduSH - *emm*58.16, tmdu302 - *emm*81, tmdu618 - *emm*28, tmdu1312-*emm*106, and tmdu1326 - *emm*106.

### GAS DNA detection by culture-independent methodology

**Fig. 5:**
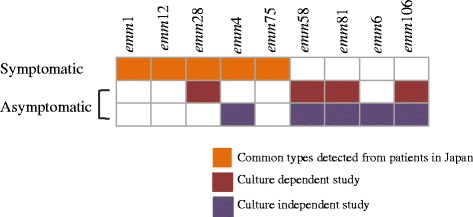
Common *emm* types associated with symptomatic cases (Japan) and *emm* types detected from healthy people. The common *emm* types found in symptomatic patients in Japan and the *emm* types detected in asymptomatic people (this study) are shown as colored boxes

Culture-independent molecular methods can detect microorganisms directly from DNA and RNA extracted from environmental and clinical samples [19, 22, 23, 35]. We used a DNA-based PCR method to gain better insight into the presence of GAS in healthy adults. As described above and shown in Fig. 4a, GAS was isolated in only 3.4 % of the samples by the culture-dependent method. In contrast, of the 148 throat swabs, GAS DNA was detected in 146 (98.6 %) samples using PCR with both 16S rRNA and V-Na^+^-ATPase primer pairs (Fig. 4b). To exclude the possibility of the primer sets cross-reacting with other DNA sequences in vivo, the homologies of 50 random 16S rRNA and V-Na^+^-ATPase amplicon sequences were confirmed by BLAST analysis against *S. pyogenes*. The 98.6 % detection rate is much higher than those of conventional standard culture methods. To determine the *emm* types, nested PCR using *emm*-conserved region primer pairs was performed following *vir* typing. Among the 146 samples, the following *emm* types were identified in 25 of them (16.9 %) by sequence analysis (Fig. 4b): *emm*58.16 (*n* = 13), *emm*106.0 (*n* = 3), *emm*81.0 (*n* = 2), *emm*6.52 (*n* = 3), and *emm*4.0 (*n* = 1) (Fig. 5, Additional file 1: Table S4). Notably, three samples (*n* = 3) with both *emm*58.16 and *emm*6.52 were detected.

## Discussion

In clinical and environmental microbiology, rapid and accurate identification of microorganisms is extremely important. Amplification of 16S rRNA and other conserved genes using species-specific primers has now been established as a promising molecular detection method. For rapid detection of GAS from patients, several species-specific primers have been designed and their efficiencies evaluated in previous studies [36–39].

Here we evaluated the use of species-specific primers to detect the presence of GAS DNA in throat swab samples from healthy adults based on culture-independent methods. We tested 148 samples using GAS-specific primers targeting 16S rRNA and V-Na^+^-ATPase genes. The ability of the 16S rRNA V2 region to differentiate common *Staphylococcal* and *Streptococcal* pathogens was demonstrated by Chakravorty et al. [15], and the V2 region was also used as a marker to identify *S. pyogenes*, *S. agalactiae* and *S. pneumoniae* in Psoriatic arthritis patients [40]. Also, a study based on simultaneous detection of the causative agent of community-acquired pneumonia demonstrated the reliability of the pathogen-specific 16S rRNA molecular beacon probe for detection of GAS [41]. In the Streptococcus genus, V-Na^+^-ATPase has only been found in *S. pyogenes* and *S. pneumoniae* [42], and Hung et al. reported the reliability of the V-Na^+^-ATPase primer set for GAS identification [27]. The 16S rRNA and V-Na + −ATPase primer sets used in the present study were validated to ensure their high specificities and sensitivities and their effectiveness at detecting the small amount of GAS DNA present in the human throat. Here, the majority of the adults tested were positive for the presence of GAS DNA (146 of 148, 98.6 %) by PCR screening. The GAS DNA carriage rate observed in this study is the highest reported for a healthy population. However, because the ‘healthy’ participants in the present study belonged to a university dental faculty they were occupationally associated with patients, making frequent exposure to GAS via respiratory droplets a possibility for them. It will be interesting for future studies to analyze samples from healthy people from non-health care settings alongside samples from health-care professionals. Nevertheless, these results suggest that culture-independent methods should be considered when seeking comprehensive understanding of the presence of GAS in healthy hosts.

To be confident about the results obtained by both detection methods (culture-dependent and culture-independent), we must contemplate why a detection rate difference was observed between them. First, asymptomatic bacterial infections are maintained by a low replication rate, which controls bacterial growth and promotes bacterial survival in hostile environments [3]. Because such populations contain small numbers of bacteria, it is difficult to isolate them from commensal-rich environmental samples, while culture-independent methods can easily detect such populations. Second, to overcome physicochemical stress in normal healthy body niches, many pathogens adapt to a VBNC state (e.g., *Vibrio cholerae* and *Escherichia coli*) [12]. Previous studies have each revealed the presence of a small subpopulation of GAS in a VBNC state under stress conditions [43, 44], and such bacteria are only traceable by culture-independent methods. Another possibility that may account for the different detection rate between the two methods is the presence of GAS in biofilms, where the hidden population might be difficult to eradicate post-infection and can remain protected from host immunity. Such populations make bacterial isolation difficult, but they can be detected easily by culture-independent methods [35, 45–47].

However, DNA-based PCR methods cannot distinguish between dead and viable bacteria, and this may lead to overestimation of bacterial numbers [48, 49]. Therefore, with culture-independent methods, we cannot rule out the presence of residual DNA from recent bacterial exposure or recurrent pharyngeal GAS carriage. To confirm the presence of an active population of asymptomatic GAS would require further evaluation based on total RNA extraction from throat swab samples or use of propidium monoazide to distinguish live from dead bacteria in DNA-based PCR methods [50]. Despite this issue, our results still shed light on GAS carriage, and the primers used in this study will be useful for future analysis of GAS prevalence.

For monitoring GAS strain diversity, an important cell surface virulence factor, the M-protein encoding *emm* gene, has been used successfully over the last two decades, because its hypervariable 5’-end makes it suitable for this purpose [31]. Therefore identification of *emm* types is important for keeping track of the clonal spread associated with GAS infections. Here, to investigate *emm*-type information, we designed a new primer pair and obtained *emm* sequence information from 16.4 % (25/148) samples. Interestingly, three samples possessed two different *emm* types, a result rarely reported [51]. In Japan, the major *emm* types associated with symptomatic GAS infections are *emm*1, *emm*12, *emm*4, *emm*28, and *emm*75 [52–55]. Here, we detected *emm*58, *emm*106, *emm*81, and *emm*6 types from healthy people. Even though *emm*58 is not commonly found in worldwide studies, there are some reports regarding this *emm* type in Japan (which is mainly associated with skin infections) and India [53–57]. These results indicate that unique *emm* types might be associated with GAS carriage in healthy people (Fig. 5).

A major drawback of the *emm* detection method was its limited specificity resulting from the *emm* and/or *emm*-like genes that are present in many streptococcal species [58], as well as the nonspecific amplification of human DNA in the samples. Although there is clearly a need to consider removing human DNA from samples to increase the specificity of this assay, we believe that our study was partially successful in obtaining *emm* information by the culture-independent method, a finding not reported in earlier PCR-based studies [36, 38–41].

## Conclusions

In this study, we investigated the presence of GAS in throat swab samples from healthy adults in Japan using culture-dependent and culture-independent methods using species-specific primers. The GAS isolation rate was consistent with previous studies; however, culture-independent detection by conserved GAS-specific primers confirmed the presence of GAS DNA in the majority of healthy adults [Fig. 4]. These results suggest that not only school-age children, but also healthy adults can serve as GAS carriers. Culture-independent methods should be considered when seeking comprehensive understanding of the presence of GAS in healthy hosts. The GAS-specific primers (16S rRNA and V-Na^+^-ATPase) used in this study can be considered useful tools for PCRs aimed at estimating the maximum potential of GAS carriage in people.

## Additional file

Additional file 1: Table S1. Samples collected from healthy adults. **Table S2.** GAS and other streptococcal isolates used for checking the specificities of all the primers used in this study. **Table S3.** The PCR conditions used for the culture-dependent and culture-independent studies. **Table S4.** The *emm* types detected in the samples, as determined by culture-independent and culture-dependent methods (XLS 48 kb)
